# Do Health-Related Quality of Life and Pain-Coping Strategies Explain the Relationship between Older Women Participants in a Pilates-Aerobic Program and Bodily Pain? A Multiple Mediation Model

**DOI:** 10.3390/ijerph16183249

**Published:** 2019-09-04

**Authors:** Pedro Jesús Ruiz-Montero, Gerardo José Ruiz-Rico Ruiz, Ricardo Martín-Moya, Pedro José González-Matarín

**Affiliations:** 1 Department of Physical Education and Sport, Faculty of Education and Social Sciences, Campus of Melilla, University of Granada, 52071 Melilla, Spain; 2 Department of Education, University of Almería, 04120 Almería, Spain; 3 Body Expression area, Education School, University of Granada, 18011 Granada, Spain

**Keywords:** aging, physical activity, pain, women’s health, mediation

## Abstract

This study (1) analyzes the differences between non-participating and participating older women in terms of clinical characteristics, pain coping strategies, health-related quality of life and physical activity (PA); (2) studies the associations between non-participants and participants, clinical characteristics, pain coping strategies, HRQoL and bodily pain and PA; and (3) determines whether catastrophizing, physical role, behavioural coping, social functioning and emotional role are significant mediators in the link between participating in a Pilates-aerobic program (or not) and bodily pain. The sample comprised 340 older women over 60 years old. Participants of the present cross-sectional study completed measures of clinical characteristics: HRQoL using the SF-36 Health Survey, pain-coping strategies using the Vanderbilt Pain Management Inventory (VPMI) and PA using the International Physical Activity Questionnaire (IPAQ). Significant differences between non-participants and participants, were found in clinical characteristics, pain-coping strategies (both, *p* < 0.05), HRQoL (*p* < 0.01), and PA (*p* < 0.001). Moreover, catastrophizing support mediated the link between non-participants and participants and bodily pain by 95.9% of the total effect; 42.9% was mediated by PA and 39.6% was mediated by behavioural coping. These results contribute to a better understanding of the link between PA and bodily pain.

## 1. Introduction

The aging population is a global phenomenon that affects all countries of the world, but especially Western countries [1]. Two billion people in the world will be over 60 by 2050, and 400 million will be over 80 [2]. Consequently, this increase in longevity contributes to the aging population. Health is one of the most important features of older people’s lives, contributing to their life satisfaction, health-related quality of life (HRQoL) and successful aging [3].

The repercussions of pain must be examined in older people because aging is associated with the risk of experiencing chronic pain [4] and psychological disorders [5]. The musculoskeletal system is severely affected by chronic pain in older people [6] and associated with disability, severity of pain or poorer HRQoL [7]. Moreover, pain-coping strategies influence the perception of pain [8]. Effects of pain are more prevalent in the context of rising life expectancy [9], whereas activities focused on health promotion and pain management in older adult patients are necessary to maintain HRQoL and avoid psychological illness or dementia [10]. HRQoL is often used to evaluate the health status of older women [11]. Predictors of HRQoL are shown as necessary to recognize the well-being of older people’s daily life, such as general health, physical role and social interactions [12]. Increasing longevity requires a better understanding of HRQoL changes and HRQoL predictors through suitable strategies to prevent long periods of morbidity and a decrease in HRQoL, resulting in a worse physical and mental health status [13]. Normally, the HRQoL scores obtained by older people in areas such as physical functioning, physical role, general health and social functioning show a decrease [14].

**Figure 1:**
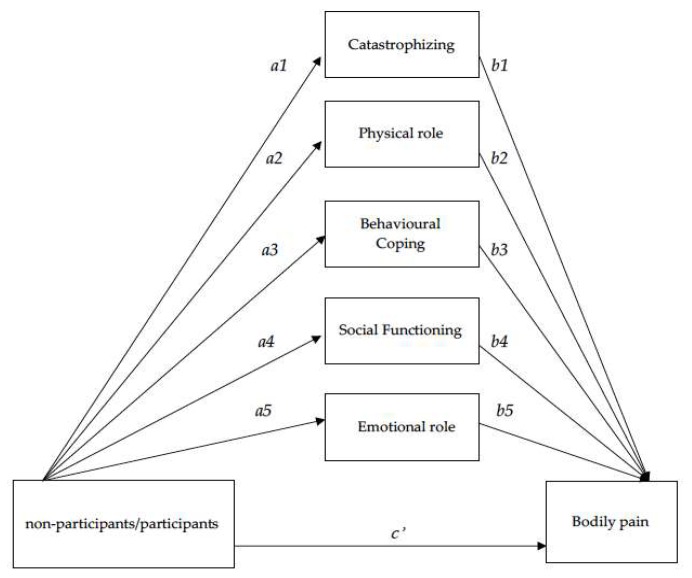
Hypothetical model of the relationship between non-participants/participants and bodily pain through catastrophizing, physical role, behavioural coping, social functioning and emotional role.

Physical activity (PA) is a reliable predictor of life expectancy and well-being in older women [15]. Well-being in older people is often not satisfactory due to health limitations such as psychological and functional problems [16]. The association between PA practice and HRQoL provides a multitude of health benefits for older people [17]. Moreover, physical inactivity and sedentary lifestyles are both causes of negative health consequences [18], such as decreasing cognitive and functional capacity [3]. Nevertheless, older people in Western countries tend to live sedentary lifestyles. In fact, although the benefits of PA on physical and functional deterioration have been thoroughly confirmed, the majority of older adults take little part in regular PA [1]. Therefore, to understand in depth the relationship of HRQoL pain-coping strategies and PA in non-participant and participant groups in a Pilates-aerobic program, the aims of this study were, first, to analyse differences between older adult women non-participants and participants in terms of clinical characteristics, HRQoL, pain-coping strategies and PA; secondly, to study the association between bodily pain, non-participants/participants, clinical characteristics, pain-coping strategies, PA and rest of HRQoL dimensions; and, thirdly, to determine the mediating role in the link between being non-participants/participants and bodily pain (Figure 1) through catastrophizing (pain-coping strategies dimensions), physical role (HRQoL dimensions), behavioural coping (pain-coping strategies dimensions), social functioning and emotional role (both, HRQoL dimensions).

## 2. Materials and Methods

### 2.1. Participants

**Figure 2:**
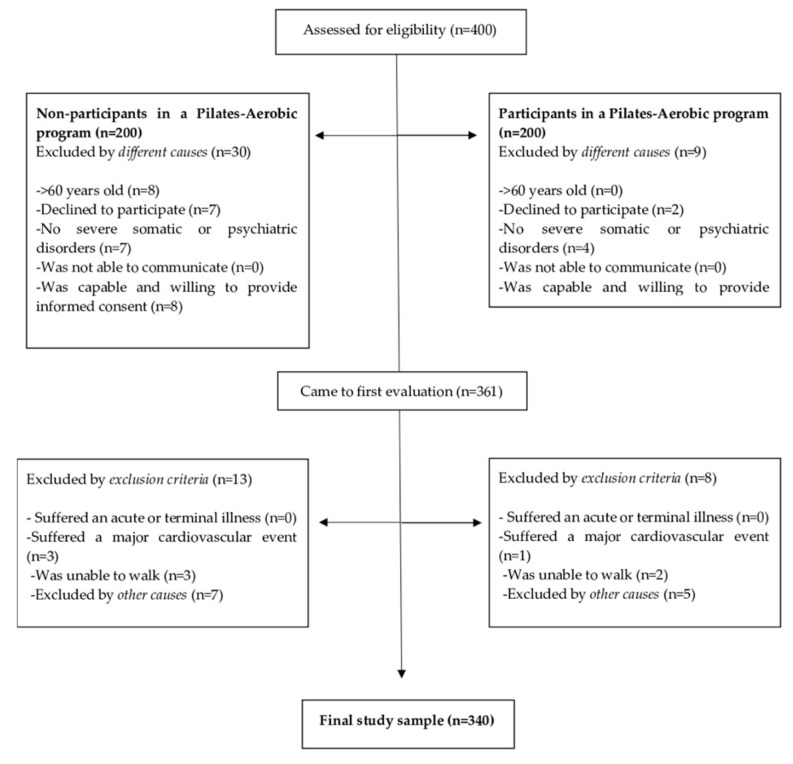
Participant’s flow diagram.

In this study, a cross-sectional design was used, and the sample was selected using a non-proportional quota sampling. The sample size was calculated at a 95% confidence level based on women >60 years old. The total sample was composed of a population consisting of 340 older women. According to the profile of the older women in the present study, 157 were non-participants in any PA program (M_age_ = 69.9, SD = 7.1) and did not practice any PA supervised by specialists, while 183 were participants and attended a Pilates-aerobic program (M_age_ = 68.8, SD = 5.3), supervised by specialists (Figure 2). Moreover, the majority of older women lived alone (non-participants = 70.8%, participants = 73.6%) and most of them did not smoke (non-participants = 93.8%, participants = 99.4%). Inclusion criteria for non-participants and participants group in a Pilates-aerobic program in this study were: (1) age >60 years; (2) no severe somatic or psychiatric disorders, or diseases that prevent physical loading; (3) able to communicate; (4) capable and willing to provide informed consent. Specific inclusion criteria for non-participants in a Pilates-aerobic program were: (1) not to be engaged in regular PA > 20 min on > 3 days/week in the last three months. Exclusion criteria for the current cross-sectional study were: (1) acute or terminal illness; (2) had suffered a major cardiovascular event (i.e., myocardial infarction, angina, or stroke) in the past six months; (3) unable to walk; (4) had an unstable cardiovascular disease or other medical condition; (5) had suffered an upper or lower extremity fracture in the past six months; (6) disinclination to complete the study requirements; and (7) the presence of neuromuscular disease or drugs affecting neuromuscular function.

This study presents two types of profile participants: (1) older women who do not practice any regular and controlled PA supervised by a specialist, considered non-participants in the present study, and (2) older women who are participants in a Pilates-aerobic program of the Málaga Provincial Government (Spain). This program was created to improve the physical fitness of older people of small and medium-sized towns of Málaga province. Both groups were recruited via telephone or direct contact (from door to door in small participant towns). It is important to highlight that the group of participants were already attending a Pilates-aerobic program in community centres when they were surveyed with the measurements cited. However, the recruitment and contact details of non-participants were obtained by collaboration of town councils.

The non-participant and participant groups in the present study were obtained by a stratified sample population approach from the villages of Malaga province with a population of between 2000 and 5000 according to the Spanish Government’s National Institute of Statistics (http://www.ine.es/).

### 2.2. Measurements

Clinical characteristics such as weight (kg), fat mass (FM) (% and kg), muscle mass (MM) (% and kg), and body mass index (BMI)(kg/m^2^) were measured by bioelectrical impedance analysis with a Tanita SC 330s. Height (cm) was measured using a stadiometer (Seca 22, Hamburg, Germany). Waist circumference (cm) was measured at the middle point between the ribs and ileac crest with the participant standing (Harpenden anthropometric tape, Holtain Ltd., Crymych, UK).

#### 2.2.1. Health-Related Quality of Life by SF-36 Health Survey (SF-36)

The Spanish version of the SF-36 Health Survey [19] was used to assess HRQoL of the older women. This questionnaire is composed of 36 items, grouped into eight dimensions and two components: physical functioning; physical role; bodily pain; general health (physical component, all); vitality; social functioning; emotional role; and mental health (mental component, all). The answer format is a yes or no alternative and a response scale from three to six. Each dimension score is ranged from 0 to 100, where 0 indicates the worst possible health status and 100 the best possible. The reliability and stability analysis of the SF-36 Health Survey showed that the correlation coefficients between test and retest were from 0.58 SF-36 emotional role to 0.99 for SF-36 physical role. Internal consistency showed an alpha coefficient between 0.78 for SF-36 vitality and 0.96 for SF-36 physical role.

#### 2.2.2. Pain-Coping Strategies by Vanderbilt Pain Management Inventory (VPMI)

The Spanish version [20] of the Vanderbilt Pain Management Inventory (VPMI) [21] was used to assess pain-coping strategies. The inventory comprises 18 items grouped in two scales (passive and active strategies). These two scales assess how often chronic pain sufferers use active and passive coping. The frequency with which patients use each strategy when their pain reaches a moderate or greater level of intensity is rated on a five-point scale. Seven of the item scores are added up to determine the active coping scale score, and the remaining 11 items are added up to determine the passive coping score. Active coping is when patients attempt to function despite their pain, and passive coping is when patients relinquish control of their pain to others or allow areas of their life to be adversely affected by pain [22]. In addition, passive and active strategies are divided into four groups respectively: catastrophizing (items 1, 2, 3, 7 and 10), social support seeking (items 4, 5, 6 and 9), behavioural coping (items 8, 12, 14, 16 and 17) and suppression (items 11, 13, 15 and 18).

#### 2.2.3. Physical Activity by International Physical Activity Questionnaire (IPAQ)

The level of PA was assessed by the International Physical Activity Questionnaire (IPAQ) for Spanish older people [23]. The IPAQ long version (31 items) was used in the present study to collect detailed information about the specific type assigned to each category of activity according to MET energy expenditure estimate over the last seven days: low, moderate and vigorous level of physical activity. MET is the calculation of energy expenditure by multiple of metabolic rate and the unit used, MET*min. MET for a specific PA is multiplied by minutes spent on that that activity per day or per week (MET*min*wk^−1^). The IPAQ has acceptable measurement properties with a repeatability coefficient of 0.81 (95% CI 0.79–0.82) [24]. The IPAQ long version data estimates total weekly physical activity by weighting the reported minutes per week [24].

### 2.3. Procedure

When the older women agreed to participate, they were given specific information questionnaires about illness, intake of pills, or disabilities to verify whether they were eligible under the inclusion and exclusion criteria. After providing information about the study (purpose, expected duration of the questionnaires interview and procedures), the informed consent form was signed. All older women (non-participants and participants of a Pilates-aerobic program) in the present study were given two days to complete all of the measurement protocols. The first day, they had to complete the following assessments in order of presentation: clinical characteristics information and body composition parameters. The next day, they filled in the questionnaires related to HRQoL (SF-36), pain-coping strategies (VPMI) and PA (IPAQ) in the same sport hall where the participants of a Pilates-aerobic program were performing the PA.

The interviewer read each question aloud and recorded the participant’s answer on the answer sheet; participants had showcards with the answer options for each scale. The contents of the program for the older women participants in a Pilates-aerobic program consisted of musical based aerobics and Pilates, basic to intermediate level [3]. It included content such as upper- and lower-body strengthening exercises and agility and aerobic capacity exercises through Pilates. Concepts of health education were given following these sessions in order to orientate participants towards a healthier posture and the practice of food hygiene in their daily lives. The sessions included in this program took place twice a week, and lasted 55–60 min each with an effective PA time of 45 min, in line with the requirements laid out by the American College of Sports Medicine [25,26]. Eighty percent of the participants completed the Pilates-aerobic program.

### 2.4. Statistical Analyses

Processing of the collected data included the use of adequate statistical methods for calculating central and dispersion parameters (arithmetic mean and typical deviation), frequency and percentage. The determination of significant differences between inactive and active participants was performed by a Mann–Whitney U test in clinical characteristics (age and body composition), VPMI, SF-36 Health Survey values and IPAQ. In order to analyse the impact of bodily pain SF-(36 dimension) on clinical characteristics, non-participants vs. participants, VPMI, the rest of the SF-36 dimensions, and IPAQ, a stepwise multiple regression analysis was performed. The linearity, independence (Durbin-Watson = Bodily pain SF-36 Health Survey dimension, between 0 and 4), normality and lack of multicollinearity (the tolerance levels > 0.1 and the variance inflation factor < 10) assumptions were met for the stepwise multiple regression analysis. The order of variables entered in the different steps was based on the study’s aims and on findings from the older people and PA literature that indicates that HRQoL is a factor that allows both healthy and symptomatic older people to cope with pain [27]. In this way, the clinical characteristics dimensions were entered in the first step and subsequently VPMI, the rest of SF-36 dimensions, and IPAQ. To test whether the association between non-participants vs. participants and bodily pain was mediated by catastrophizing, physical role, behavioural coping, social functioning and emotional role, a linear regression model was fitted using bootstrapped mediation procedures [28]. Absence of collinearity assumptions (using correlation matrix, VFI and eigenvalues) were tested.

Data were analysed using the SPSS statistical program (IBM SPSS Statistics for Windows 21.0. Armonk, NY, USA). For all analyses, significance was accepted at *p* < 0.05.

## 3. Results

Clinical characteristics of the study sample, VPMI dimensions and SF-36 Health Survey dimensions are specified by non-participants and participants group in Table 1. Clinical characteristics such as body weight (*p* < 0.01), BMI (Kg/m^2^), fat mass (kg) and muscle mass (kg) (all, *p* < 0.05) showed differences. According to VPMI dimensions, the participant group showed higher behavioral coping and active strategies scores (both, *p* < 0.05) and lower social support seeking (*p* < 0.001) and passive strategies scores (*p* < 0.05) than the non-participant group. Moreover, SF-36 Health Survey dimensions such as physical functioning, bodily pain dimensions (*p* < 0.001), and physical role (*p* < 0.01) in the participant group showed higher scores than the non-participant group. However, the non-participant group showed higher general health, vitality and mental health (*p* < 0.001) than the participant group. Finally, the participant and non-participant groups showed differences in PA (*p* < 0.001).

In the stepwise multiple regression, catastrophizing (31%), physical role (42%), behavioural coping (49%), social functioning (50%) and emotional role (51%) were significant predictors of the variance in bodily pain, as Table 2 conveys.

The multiple mediation analysis using the 5000 bootstrap and bias-corrected and accelerated 95% CI [28] revealed that the paths from being non-participants/participants to catastrophizing, physical role and behavioural coping were significant (Table 3). The non-participants/participants variable was positively associated with catastrophizing, physical role (both, *p* < 0.05) in the first regression equation. In the second equation, catastrophizing, physical role, emotional role (all, *p* < 0.001) and behavioural coping (*p* < 0.01) were positively associated with bodily pain, while social functioning was not statistically significant. Finally, in the third equation, when the non-participants/participants variable and mediators were simultaneously included in the model, there was positive association between non-participants/participants and behavioural coping (*p* < 0.001), social functioning, emotional role (both, *p* < 0.01), catastrophizing and physical role (both, *p* < 0.05).

The mediation estimated that 95.9% of the total effect of the non-participants/participants variable on bodily pain was mediated by catastrophizing, 42.9% was mediated by physical role, and 39.6% was mediated by behavioural coping.

## 4. Discussion

The aims of the present study were, first, to analyse differences between female non-participants and participants in a Pilates-Aerobic program in terms of clinical characteristics, HRQoL, pain-coping strategies and PA. The second aim was to study the association between bodily pain, non-participants/participants, clinical characteristics, pain-coping strategies, PA, and the rest of the HRQoL dimensions. Finally, the third aim was to determine the mediator’s role in the link between being non-participants or participants and bodily pain (Figure 1) through catastrophizing, behavioural coping (pain-coping strategies dimensions) and physical role, social functioning and emotional role (HRQoL dimensions).

With regards to clinical characteristics of the participants, evidence from this study suggests differences in body composition between the non-participant and participant groups in a Pilates-aerobic program. The greater content of muscle mass percentage and body weight may be due to the influence of strength-resistance training the with Pilates method [29]. In addition, Pilates exercise improves more synthesis of muscle proteins and, therefore, a greater muscle mass content; it also increases bone mineral density in the body composition [30]. It is important to highlight that the percentage of muscle mass is lower in the older population than in adults [31]. In fact, strength-resistance training allows an increase in muscle mass content. On the other hand, as a consequence of the resistance training, there is a greater energy expenditure where the use of fat during this type of exercise is moderate [32]. Moreover, exercise resistance increases the basal metabolic rate, which allows for greater daily energy expenditure [33]. For this reason, members of the participant group have a lower percentage of fat mass and BMI than the non-participants group.

According to the differences in HRQoL in both groups, one of the reasons that the non-participant group presents better vitality and general health than the participant group might be that they engage in enough PA in their daily life and it is not necessary to participate in a Pilates-aerobic program that complements the daily PA done. Since one of the motivations to perform a continuous program of PA is the need to perform it to improve their health [34], the participant group could have decided to take part in the Pilates-aerobic program to improve their general health and daily PA.

The dimension of mental health (anxiety, depression and general well-being level) is higher in the non-participant group than the participant group, and this can be explained because one of the motivations for the participants to engage in Pilates-aerobic exercise was because of symptoms of anxiety or depression [35], and they were looking to improve their personal situation by carrying out a specific PA program. On the other hand, one of the reasons that the non-participant group did not perform the Pilates-aerobic program was because they felt their mental health status was better and did not feel the need to participate in any supervised PA [36]. Therefore, non-participants in our study showed better perception in several HRQoL dimensions, confirming previous studies in which physically inactive participants had a better perception of their general health than active participants in a PA program [37].

People who do not suffer from any type of chronic pain are more likely to perform some type of PA. In addition, if PA or physical exercise is performed, it may reduce the sensation of bodily pain [38]. In a study by Vincent, George, Seay, Vincent and Hurley [39], in which 49 older obese people, mostly women with low back pain, participated in a resistance exercise program, it was found that with the improvement of physical functioning, specifically physical resistance, passive strategies of pain control were considerably reduced. In addition, a decrease in participants’ perceived disability caused by pain could also be observed. Structured PA enhances HRQoL and perceived physical functioning [40]. Thus, it is reasonable to believe that through PA, physical function and physical role will improve [41].

Regarding the assessment of pain-coping strategies, participants showed lower levels of social support seeking because they might experience high interrelationship and support while practicing PA. This is due to the social component of sport practice being one of the greatest benefits of doing a PA program [42]; moreover, doing PA reduced pain perception, with the major release of endorphins, leading to higher levels of trust and personal autonomy [43]. In addition, the women in the participant group reported a higher behavioural coping than the non-participant group. The regular practice of PA decreases the sensation of pain and, as a consequence, allows them to perform daily life activities in the best way [44].

Thus, passive strategies have shown higher levels in the non-participant group, which could be a sign that they do not try to find options to cope with the pain they feel in their daily life [45]. One of the reasons could be the difficulty older people in rural areas have with accessing information and resources [46], although the reasons some people engage in active coping while others do not remain unclear [47].

To the authors’ knowledge, the present study is the first to moderate several VPMI and SF-36 dimensions between non-participant and participant groups in a Pilates-aerobic program and bodily pain in a studied sample.

One of the associations that we found in our research is the mediating role of catastrophizing among the women participating in the present study and their corporal pain. This finding is in line with other studies, such as the one carried out by Ohlman et al. [48], in which a program of isometric exercises was applied to older people. This study showed that, with the implementation of this program, the level of participants’ catastrophism decreased. As we can understand, on the one hand, this variable can limit people while practicing PA [49], but once they perform PA, the level of catastrophism can decrease [48]. Accordingly, Marshall, Schabrun and Knox [50], in their study of 154 participants of advanced age, observed that there was a significant decrease of catastrophism perceived following sports and PA practice. Consequently, these authors affirm that the mediation between level of catastrophism and sports practice may be due to the fear that these people may have of experiencing pain during the performance of PA.

On the other hand, reducing the practice of PA can have a great impact on bodily pain [51]. Although some studies contradict this theory [52,53], other studies clearly affirm that adequate practice of PA helps to decrease bodily pain and, therefore, to improve levels of catastrophism [54,55]. A clear example is the study carried out by Ardoin and Canot [56] in which a program of sports activities adapted to people suffering from chronic pain was applied. It was shown that the subjects who participated in this program decreased their bodily pain and levels of catastrophism, causing an improvement in their HRQoL.

If we observe interventions like ours, developed only by older women, we can see a clear decrease in catastrophism with the implementation of PA, which led them to perceive a lower pain sensation [57]. A study conducted by Scurati et al. [58], whose participants, like ours, were older women, showed how through an adequate aerobic activity program in an aquatic environment, it was possible to reduce their perception of pain. Therefore, according to the study mentioned above, it is evident that older women who perform PA can decrease their level of catastrophism and, consequently, their bodily pain as well.

The results of the present study also provide evidence for the mediating role played by the physical roles of the participant and non-participant groups related to the bodily pain they experience. This statement is very logical if we observe, on the one hand, the different interventions that affirm that through an adequate PA it is possible to improve the different physical aptitudes and improve the performance of daily activities [59], and the several studies that affirm that this improvement of the physical role of the subject affects a decrease in their bodily pain [60]. The relationship between these three variables can be observed in earlier research [61]. A clear example is the intervention program carried out by Pazit et al. [62] in which, through the application of a resistance and balance training program, it was possible to appreciate the improvement of the physical aptitudes of the participants in day-to-day activities and, on the other hand, the reduction of their bodily pain. However, if it is not performed appropriately, the relationship between these variables may not exist [63].

Behavioural coping was also a mediating factor between the participant and non-participant groups in this study and their bodily pain. In a study that precedes ours, it can be highlighted the use of a good coping strategy and the realization of PA when it comes to overcoming a pain or diminishing it [64]. In our opinion, it is possible that one of the most important mediating effects of the behavioural coping variable between the participants group of our study who performed PA and their bodily pain was related to adherence to PA, due to the palliative effects on generalized fatigue, physical problems and HRQoL [65]. In this context, it is interesting to mention the review study through meta-analysis developed by Nicolson et al. [66], in which the importance of adherence to PA is evident in people with chronic low back pain when overcoming this pain barrier. Several interventions are shown in which, through a program of adequate PA and establishment of behavioural-based coping strategies based on motivation, it is possible to increase adherence to PA.

## 5. Conclusions

The outcomes obtained in the present study show how engaging in a Pilates-aerobic program helps older women cope with pain. That was not the case with HRQoL, where the non-participant group experienced better feelings than the participant group in several dimensions. There are some limitations of the present study which affect the generalizability of the findings. The results of the regression analysis and of the multiple mediation analysis should be interpreted cautiously due to the cross-sectional design that does not allow for distinguishing between predictors and outcome variables. It is also possible that bodily pain or the extent to which an individual has difficulties in performing daily life or work-related activities because of an impaired health status, could affect an individual´s pain coping strategies and her PA level. Therefore, future intervention studies should use longitudinal designs to determine the potential effect of participating in a PA program on HRQoL, specifically regarding bodily pain, in older women. Moreover, the use of non-probability sampling and the fact that the sample was collected from rural settings and only women took part, might not allow extrapolating the results of this study to the general Spanish population of older people. However, despite these limitations, this study contributes to a better understanding of the processes that promote life satisfaction maintenance in old age and emphasizes the coping strategies that older women could count on when they deal with health impairment and chronic pain.

To this end, it is important to support existing resources, such as senior activity centres, older people’s associations, or higher education for older people, and develop new initiatives that allow older people to engage in PA programs with specific content such as Pilates and aerobics. At the same time, it is necessary to inform families and professionals working with older people about the needs and expectations regarding social relations and PA preferences.

## Figures and Tables

**Table 1:** Differences of clinical characteristics, VPMI, HRQoL and IPAQ between non-participants and participants groups in a Pilates-aerobic program.

Variables		Participants (*n* = 183)	Non-Participants (*n* = 157)	*p*-Value ^a^
		Clinical Characteristics		
		Mean ± SD	Mean ± SD	
Age	(years)	68.85 ± 5.37	69.92 ± 7.15	0.298
Body weight	(kg)	71.77 ± 12.78	71.16 ± 12.62	<0.01
Body height	(cm)	152.31 ± 12.96	150.38 ± 13.62	0.864
Waist circumference	(cm)	100.14 ± 12.92	100.26 ± 12.16	0.284
BMI	(kg/m^2^)	30.48 ± 5.16	31.12 ± 5.15	<0.05
Fat mass	(%)	43.71 ± 10.13	44.77 ± 5.83	<0.05
Fat mass	(kg)	31.41 ± 9.31	31.72 ± 9.15	0.853
Muscle mass	(%)	30.58 ± 5.01	30.31 ± 6.29	0.149
Muscle mass	(kg)	21.95 ± 3.64	21.53 ± 4.53	<0.05
		Vanderbilt Pain Management Inventory (VPMI)		
Catastrophizing		9.27 ± 3.44	10.11 ± 4.01	0.09
Social Support Seeking		6.49 ± 2.37	7.38 ± 2.41	<0.001
Behavioural Coping		11.71 ± 3.03	11.14 ± 2.73	<0.05
Suppression		10.26 ± 3.01	9.65 ± 2.92	0.067
Passive strategies		21.13 ± 6.07	22.88 ± 6.73	<0.05
Active strategies		16.69 ± 4.53	15.6 ± 4.79	<0.05
		HRQoL (SF-36 Health Survey)		
Physical functioning (0–100)		66.11 ± 25.13	55.11 ± 25.82	<0.001
Physical role (0–100)		57.05 ± 44.57	44.12 ± 44.79	<0.01
Bodily pain (0–100)		56.15 ± 27.57	45.46 ± 29.18	<0.001
General health (0–100)		46.07 ± 19.37	56.54 ± 17.88	<0.001
Vitality (0–100)		44.48 ± 21.15	53.93 ± 22.66	<0.001
Social Functioning (0–100)		55.28 ± 17.07	56.55 ± 21.48	0.335
Emotional role (0–100)		62.57 ± 45.13	55.00 ± 47.63	0.185
Mental health (0–100)		36.48 ± 20.53	46.88 ± 22.12	<0.001
		Physical Activity Level Questionnaire (IPAQ)		
		*n* (%)	*n* (%)	<0.001 ^a^
Low		60 (32.8)	79 (50.3)	
Moderate		26 (14.2)	23 (14.6)	
Vigorous		97 (53)	55 (35.1)	

^a^*p* values calculated by U Mann–Whitney test between non-participants and participants in a Pilates-aerobic exercise program in Clinical characteristics, pain-coping strategies by VPMI, HRQoL by SF-36 Health Survey and PA level by IPAQ; values expressed as mean (SD) in clinical characteristics, VPMI, SF-36 Health Survey and *n* = number (% = percentage) in IPAQ.

**Table 2:** Results of the stepwise multiple regression analysis for bodily pain with non-participants/participants, clinical characteristics, VPMI dimensions, rest of SF-36 dimensions and IPAQ.

	Step	Variable	*R*	*R* ^2^	Adjusted *R*^2^	Standard Error	*p*-Value
Bodily pain	1	Catastrophizing (VPMI)	0.562	0.316	0.313	23.99	<0.001
	2	Physical role (SF-36)	0.653	0.427	0.422	22.01	<0.001
	3	Behavioural Coping (VPMI)	0.709	0.503	0.493	20.61	0.004
	4	Social Functioning (SF-36)	0.718	0.516	0.504	20.38	0.011
	5	Emotional role (SF-36)	0.724	0.524	0.511	20.24	0.038

**Table 3:** Indirect effect of non-participants/participants on bodily pain through catastrophizing, physical role, behavioural coping, social functioning and emotional role.

Mediator	Effect of X on M (a^1^-a^5^)	SE	Effect of M on Y (b^1^-b^5^)	SE	Bootstrap Estimate	SE	BCa 95% CI	
							Lower	Upper
Catastrophizing	−0.957 *	0.442	−4.153 ***	0.363	3.974	1.897	0.487	7.788
Physical role	13.051 *	5.101	0.333 ***	0.031	4.354	1.741	1.141	8.031
Behavioural Coping	0.576	0.343	−1.663 **	0.556	−0.959	0.629	−2.539	−0.013
Social Functioning	−1.675	2.111	0.111	0.086	−0.185	0.335	−1.279	0.233
Emotional role	7.545	5.275	0.263 ***	0.032	1.987	1.433	−0.787	4.885

Regression coefficients were significant at * *p* < 0.05, ** *p* < 0.01, *** *p* < 0.001; (a^1^-a^5^) = Relationship between non-participants/participants and the five mediators; (b^1^-b^5^) = Relationship between the five mediators and bodily pain.
